# Metal ion-crosslinking multifunctional hydrogel microspheres with inflammatory immune regulation for cartilage regeneration

**DOI:** 10.3389/fbioe.2025.1540592

**Published:** 2025-01-28

**Authors:** Zhuoming Xu, Jun Ma, Hanyin Hu, Jintao Liu, Haiyang Yang, Jiayi Chen, Hongwei Xu, Xinyu Wang, Huanhuan Luo, Gang Chen

**Affiliations:** ^1^ Jiaxing University Master Degree Cultivation Base, Zhejiang Chinese Medical University, Hangzhou, China; ^2^ Department of Orthopaedics, Jiaxing Key Laboratory of Basic Research and Clinical Translation on Orthopedic Biomaterials, The Second Affiliated Hospital of Jiaxing University, Jiaxing, China; ^3^ Department of Radiology, The Second Affiliated Hospital of Jiaxing University, Jiaxing, China

**Keywords:** hydrogel microsphere, magnesium ion, cartilage regeneration, osteoarthritis, anti-inflammatory, immunomodulation

## Abstract

**Introduction:**

Osteoarthritis (OA) is a degenerative disease of the joints characterized by cartilage degradation and synovial inflammation. Due to the complex pathogenesis of OA, multifaceted therapies that modulate inflammatory and immune microenvironmental disturbances while promoting cartilage regeneration are key to control the progression of OA.

**Methods:**

Herein, a multifunctional nanoparticle (DIC/Mg-PDA NPs) was constructed successfully by the metal chelation effect between Mg^2+^ and catecholamine bond from dopamine, followed by the amidation with diclofenac (DIC), which was then prepared into an injectable hydrogel microsphere (DIC/Mg-PDA@HM) with immune-regulating and cartilage-repairing abilities through microfluidic technology for the treatment of osteoarthritis.

**Results and discussion:**

The sustained release of Mg^2+^ from the composite hydrogel microspheres achieved inflammatory immune regulation by converting macrophages from M1 to M2 and promoted cartilage regeneration through the differentiation of BMSCs. Moreover, the enhanced release of DIC and polydopamine (PDA) effectively downregulated inflammatory factors, and finally achieved OA therapy. In addition, *in vivo* MRI and tissue section staining of OA model proved the significant efficacy of the hydrogel microspheres on OA. In conclusion, these novel hydrogel microspheres demonstrated a promising prospect for multidisciplinary repairing of OA.

## 1 Introduction

Osteoarthritis (OA) is a common degenerative joint disease that affects millions of middle-aged and elderly people worldwide (Lu et al., 2023; Li Z. et al., 2024; Liang et al., 2024). OA is often accompanied by an increase in intra-articular inflammatory mediators (such as IL-1β, IL-6, 15, 17, 18, 21, 22, and TNF-α, etc.) and reactive oxygen species (ROS) (Mukherjee and Das, 2024; Xue et al., 2024), which disrupts the immune microenvironment within the joint, leading to synovitis and cartilage degradation, inducing the formation of bone spurs and finally significantly affecting the patient’s life (Fernandes et al., 2020; Sanchez-Lopez et al., 2022; Zhu et al., 2024). Currently, most treatments for OA aim to relieve pain and improve joint function (Katz et al., 2021). Traditional surgical treatments, such as total joint replacement, involve large incisions and a range of postoperative complications (Wu et al., 2022). Systemic oral medication treatments, such as non-steroidal anti-inflammatory drugs (NSAIDs), are in low bioavailability and severe gastrointestinal side effects (Richard et al., 2023; Neogi et al., 2024). Therefore, there is an urgent need to develop innovative treatment strategies that comprehensively address the changes in the inflammatory immune microenvironment and cartilage defect of joints during the progression of osteoarthritis (Huang et al., 2023; Jiang et al., 2023; Paesa et al., 2023).

Various metal ions have been widely used in tissue engineering for bone and cartilage repairing (He et al., 2023; Qin et al., 2024). Mg^2+^, as the second most abundant metal cations in the human body, is known for its superb immunomodulation, cell migration regulation, and bone regeneration abilities (Qiao et al., 2021; Shah et al., 2024; Sun Q. et al., 2024). Currently, it has been reported that an appropriate concentration of Mg^2+^ can enhance cell adhesion and promote cartilage differentiation by recruiting mesenchymal stem cells through the activation of the PI3-AKT signaling pathway, and thus induces the enhanced expression of HIF-1α (Yao et al., 2019; Yue et al., 2019; Zhao et al., 2022). Gao et al., successfully prepared magnesium gradient-based scaffolds for osteochondral defect repairing, demonstrating that different concentrations of Mg^2+^ exhibit different tendencies in bone or cartilage regeneration (Gao et al., 2023). Similarly, Liao et al. found that incorporating magnesium ions into extracellular matrix (ECM) bioactive scaffolds could effectively promote articular cartilage regeneration and modulate the polarized state of macrophages (Liao et al., 2023). However, the sole application of Mg^2+^ for OA cartilage repairing is in relatively limited efficacy (Yao et al., 2019). Additionally, due to the influence of synovial fluid in the joint cavity, Mg^2+^ will be cleared quickly within a short period, which makes it difficult to achieve a long-lasting therapeutic effect (Xiong et al., 2021). Therefore, exploring a novel drug delivery system is of great significance for OA therapy.

At present, there are many types of metal-based carriers, such as metal-organic frameworks (MOFs), metal-protein nanoparticles, and metal alloy scaffolds, which have achieved good applications in biomedical engineering (Nastri et al., 2019; Li et al., 2021; Akbarzadeh et al., 2024). However, the biotoxicity of MOFs in high concentrations, structural susceptibility of metalloproteins, and relatively large size of metal alloy scaffolds have limited their applications (Abadi and Irani, 2023; Khafizova et al., 2023; Wong et al., 2024). In contrast, it has been reported that polydopamine (PDA), due to its excellent biocompatibility, biodegradability, metal chelating ability, strong near-infrared (NIR) absorption, and ROS scavenging capability, has garnered significant attention in various fields such as biosensing, bioimaging, and drug delivery (Wang et al., 2020). The catecholamine bond from PDA can achieve chelation effect with metal ions for bulk loading, moreover, the low toxicity and nano-size allow for high-dose injection of its therapy (Wang et al., 2020; Xu et al., 2022; Yu et al., 2023). In addition, PDA can also play an important role in ROS scavenging by improving the efficiency of oxidative phosphorylation, thereby reducing the occurrence of oxidative stress (Bao et al., 2023). Thus, it would be interesting to explore the polydopamine nanoparticles chelating magnesium ions in OA therapy (Hu et al., 2023).

Intra-articular injection of drugs has gradually become a popular conservative method for OA therapy (Yao et al., 2023). However, synovial fluid often leads to rapid dilution and metabolism of the injected drugs, which makes it in poor bioavailability, in addition, multiple injections pose a risk of infection (Huang et al., 2022; Sun Y. et al., 2024). In contrast, hydrogel microspheres have been widely used as an efficient drug delivery system for tissue engineering because of their excellent biocompatibility, injectability, and modular combinability (Li et al., 2023). Properly sized hydrogel microspheres can efficiently avoid the clearance of the medicine in the joint cavity and provide a good retardation of drug release (Li and Wu, 2023). Han et al., prepared injectable hydrogel microspheres (GelMA@D-MA-MPC) with enhanced lubrication and controlled release behavior (Han et al., 2021). Therefore, it would be a promising way to develop an efficient intra-articular drug delivery system which combined hydrogel microspheres with functional nanocarriers in OA therapy (Lin et al., 2024).

**SCHEME 1:**
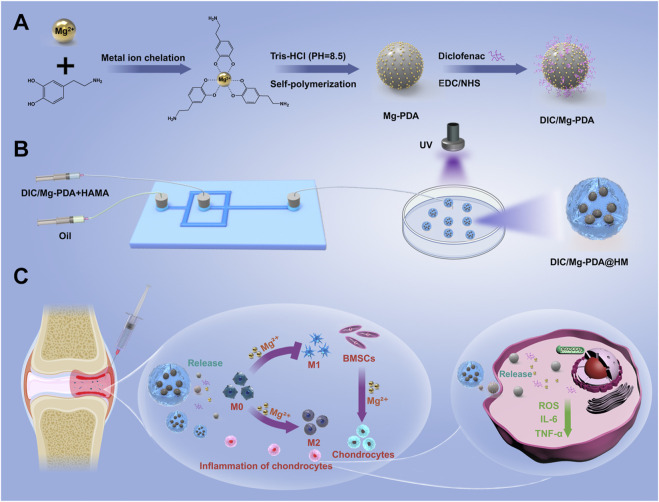
Schematic diagram of the preparation of hydrogel microspheres and osteoarthritis treatment. **(A)** Preparation of DIC/Mg-PDA nanoparticles. **(B)** Preparation of DIC/Mg-PDA@HM hydrogel microspheres. **(C)** Mechanism of DIC/Mg-PDA@HM for the treatment of OA.

Herein, an injectable hydrogel microsphere which crosslinked with multifunctional polydopamine nanoparticles was designed by microfluidic technique for cartilage repairing and immune-inflammatory microenvironmental modulation in OA. As shown in Scheme 1A, magnesium ions were first crosslinked with the catechol moiety of dopamine by the metal ion chelation, and the nanoparticles (Mg-PDA NPs) were self-polymerized under alkaline conditions (Liu S. et al., 2023). Subsequently, the non-steroidal anti-inflammatory drug (NSAID) diclofenac (DIC) was conjugated to the surface of the nanoparticles through amidation. Finally, the above nanoparticles (DIC/Mg-PDA) were doped into the hyaluronic acid hydrogel matrix as a secondary structure and crosslinked under ultraviolet (UV) light to form hydrogel microspheres (DIC/Mg-PDA@HM) via microfluidic technology (Scheme 1B). On the one hand, the DIC and PDA released from the hydrogel microspheres can effectively resist oxidative stress and downregulate inflammatory factors. On the other hand, sustained release of magnesium ions not only induced the chondrogenic differentiation of mesenchymal stem cells, but also induced transformation of macrophages from M1 to M2 phenotype. *In vivo* experiments, hydrogel microspheres were injected into the diseased joints of OA rats, and the findings based on MRI and histological staining of tissue specimens revealed their OA-relieving and cartilage-repairing effects (Scheme 1C). Thus, the novel hydrogel microspheres provide a new strategy for the integrated and comprehensive treatment of OA.

## 2 Materials and methods

### 2.1 Synthesis of Mg-PDA and DIC/Mg-PDA

As previously described, 45 mg of dopamine hydrochloride powder (Macklin, China) and 0.95 mg of anhydrous magnesium chloride powder (Macklin, China) were first dissolved in 1.5 mL of sterile water and then stirred overnight to form a dopamine-magnesium chelate solution (Liu S. et al., 2023). Take 0.45 mL of 1 M Tris-HCl (pH = 8.5) (Macklin, China) and dilute it with distilled water to a total volume of 45 mL to obtain a 0.1 M Tris-HCl solution. At the same time, add 5 mL of anhydrous ethanol (Macklin, China) to form a mixed solution. Then 0.3 mL of dopamine-magnesium chelate solution was added and the reaction was stirred overnight. Finally, the solution was centrifuged at high speed (12,500 r/min) to isolate the Mg-PDA nanoparticles, which were then washed three times with deionized water and lyophilized at −80°C.

The loading of DIC followed the reported method, 10 mg of hydrophobic DIC powder (Macklin, China) was poured into 10 mL of solution (methanol:water = 2:8) and mixed with EDC/NHS (Macklin, China) (5% w/w) for carboxylate activation (Chen et al., 2023). Then, 10 mg of Mg-PDA nanoparticles were placed into the above DIC mixture solution and a homogeneous mixture was obtained by sonication for 30 min. Next, the mixture solution was placed at room temperature and stirred for 24 h. Finally, the solution was separated by high-speed centrifugation (12,500 r/min) to obtain DIC/Mg-PDA nanoparticles and washed twice with methanol and twice with deionized water, and lyophilized at −80°C.

### 2.2 Characterization of Mg-PDA and DIC/Mg-PDA

The morphology of Mg-PDA and DIC/Mg-PDA were recorded by TEM (Tecnai-G20), and the size of nanoparticles and the zeta potential were measured by Zetasizer Nano ZSE (Malvern). In addition, the elemental composition and distribution were determined by means of EDS (Xplore30). We performed FTIR (Thermo Nicolet iS5) to analyzed the structure of the materials (spectral range from 400 to 4,000 cm^−1^). Finally, we determined the UV absorbance of each sample separately by a microplate reader (Multiskan GO).

### 2.3 Synthesis of hydrogel microsphere (HM) and hybrid HMs

The synthesis of HM was prepared by a microfluidic device. First, 200 mg of HAMA (EFL-HAMA-150K) and 10 mg of photoinitiator lithium phenyl-2,4,6-trimethylbenzoylphosphinate (LAP) completely dissolved in 10 mL of PBS under sonication for 30 min, which was used as the aqueous phase. Then, 0.5 mL Span80 and 9.5 mL mineral oil were mixed as the oil phase. The microfluidic device was used to make the two phases co-flow in the microfluidic droplet chip according to a certain proportion of the flow rate to form droplets with uniform particle size. Next, the flowing HM droplets were cross-linked under ultraviolet irradiation to obtain coarse HM solid particles. Finally, the cured HM was washed thoroughly with 75% ethanol and lyophilized at −80°C. To prepare hybrid HMs (i.e., DIC/Mg-PDA@HM, DIC@HM, Mg-PDA@HM), we completely dissolved different samples (i.e., DIC/Mg-PDA, DIC, Mg-PDA) into the HAMA and photoinitiators solutions as the aqueous phase, respectively, and other steps were the same as those for HM synthesis.

### 2.4 Characterization of HM and DIC/Mg-PDA@HM

The morphology of microspheres prepared by the microfluidic device at different aqueous and oil phase flow rate ratios were imaged under a microscope. The morphology of lyophilized HM and hybrid HM was examined by SEM (HITACHI SU8010). FTIR was also used to analyze the chemical structures in hydrogel microspheres. In addition, in order to determine the distribution of the DIC/Mg-PDA NPs in the hydrogel microsphere, the fluorescent chromogenic agent indocyanine green (ICG) was loaded into the nanoparticles as a marker for tracing. And the tagged HMs was imaged using a fluorescence microscopy.

### 2.5 Measurement of drug loading and release behavior

The DIC drug loading capacity (DLC) of DIC/Mg-PDA@HM was obtained by a microplate reader (Multiskan GO) at 280 nm. DLC was calculated according to the equation.

$$\text{DLC }\left(\%\right)=\left(w_{1}-w_{2}\right)/w_{3}\times 100\%$$

where w_1_ stands for the total quality of drug input, w_2_ stands for the total quality of drug in the supernatant during drug loading, and w_3_ stands for the total quality of carrier and drug.

The release of metal ions and drug DIC was determined by wrapping 1 mL of DIC/Mg-PDA@HM and DIC/Mg-PDA solution in a dialysis membrane bag (MWCO 12 000), soaking it in 5 mL of PBS solution. Afterwards, placing it in a rotator at 37°C (60 rpm). At specified intervals, 2 mL of the receiver solution was gathered and poured in 2 mL fresh PBS. And then, the receiving solution gathered at each designated moment was assayed for released DIC and Mg^2+^ by a microplate reader or an inductively coupled plasma emission spectroscopy (Agilent 5110), respectively.

### 2.6 Degradation behavior

The degradation of HM and DIC/Mg-PDA@HM were examined by dispersing them in a collagenase I solution which simulated a physiological microenvironment. Briefly, 4 mg of HM and 4 mg of DIC/Mg-PDA@HM were suspended in 1 mL 0.1% type I collagenase solution, respectively, and then placed them in a rotator at 37°C (60 rpm). At different time points, the samples from each group were collected and then lyophilized. Finally, the residual weights of HM and DIC/Mg-PDA@HM were accurately measured and respectively, compared to their initial weights.

### 2.7 Live/dead stain detection

In this study, ATDC5 cells (CL-0856) were purchased from Pricella, China. In 6-well plates, ATDC5 cells were first plated at a density of 1 × 10^6^ cells per well and incubated at 37°C and 5% CO_2_ overnight. Subsequently, PBS, HM, and DIC/Mg-PDA@HM were added into the plates. After incubation for 24 h, 48 h, 72 h, the cells were washed thoroughly with PBS and incubated with live/dead staining dyes, followed by the determination via a fluorescence microscopy.

### 2.8 Cell viability assay

We used MTT assay for cell viability determination. Similarly, ATDC5 cells were plated in 96-well plates at a density of 5 × 10^3^ cells per well at 37°C and 5% CO_2_ overnight. MTT method was used to accurately determine the OD value of each group of cells on each day using microplate reader, and finally the results were collected to analyze the cell viability.

### 2.9 Cellular uptake

In order to verify *in vitro* cellular uptake of composite hydrogel microspheres, we synthesized hybrid HM marked by ICG probes. ATDC5 cells were inoculated in 6-well plates at a density of 1 × 10^6^ cells per well for 24 h at 37°C and 5% CO_2_, and then the above hybrid HM was added into the plates. After co-culture for 6 and 24 h, washed the cells thoroughly with PBS and incubated with Hoechst 33342 at 37°C, respectively. And then the cell were washed thoroughly with PBS and imaged under a fluorescence microscope. Finally, analyzed the results using ImageJ software.

### 2.10 *In vitro* cellular ROS clearance

DHE stain was utilized to detect the level of cellular ROS *in vitro*. ATDC5 cells were inoculated in 6-well plates at a density of 1 × 10^6^ cells per well at 37°C and 5% CO_2_ overnight. After co-culture with LPS (5 ng/mL) overnight to mimic the inflammation microenvironment of ATDC5 cells, the cells were then treated with the addition of PBS, HM and DIC/Mg-PDA@HM, respectively. Subsequently, cells were washed thoroughly with PBS and stained with DHE stain for 30 min. Then, cells were washed thoroughly with PBS and imaged under a fluorescence microscope. Finally, analyzed the results using ImageJ software.

### 2.11 *In vitro* detection of inflammatory factors

The inflammatory microenvironment of ATDC5 cells in OA *in vitro* was also mimicked by adding LPS (5 ng/mL) together for 24 h incubation, as described previously. Then PBS, HM, DIC@HM, Mg-PDA@HM and DIC/Mg-PDA@HM were added and incubated with cells overnight, respectively. Subsequently, the supernatants of each group were collected and the levels of IL-6 and TNF-α secreted by ATDC5 cells were determined by ELISA kit, respectively.

### 2.12 *In vitro* chondrogenic differentiation

In this study, BMSCs cells (CP-R131) were purchased from Pricella, China. BMSCs cells were inoculated on cell slides in 24-well plates at a density of 1 × 10^4^ cells per well and cultured at 37°C and 5% CO_2_ overnight. Then, PBS, HM and DIC/Mg-PDA@HM were added for co-cultivation, respectively, and the medium was refreshed every 2 days. After incubation for 7 days, 4% paraformaldehyde was used to immobilize BMSCs cells and then cells were incubated with 0.1% Triton X-100 for 20 min, subsequently, the blocking with 5% BSA/PBS. Then, an anti-collagen II antibody was used to incubate with cells at 4°C overnight. Next, cells were washed thoroughly with PBST, followed by co-incubation with goat anti-rabbit IgG H&L for 1 h. And then cells cocultured with DAPI (4,6-diamidino-2-phenylindole dilactate) and phalloidin, respectively. Finally, imaged under a fluorescence microscope and analyzed the results using ImageJ software.

### 2.13 *In vitro* differentiation of macrophage RAW264.7 cells

In this study, RAW264.7 cells (CL-0190) were purchased from Pricella, China. RAW264.7 macrophages were inoculated on cell slides in 24-well plates at a density of 1 × 10^4^ cells per well and incubated at 37°C and 5% CO_2_ for overnight. It was followed by incubation with LPS (5 ng/mL) for 24 h to mimic the immune microenvironment disorders *in vitro*. Then, PBS, HM and DIC/Mg-PDA@HM were added and co-incubated for another 24 h, respectively. The following steps were performed as before, 4% paraformaldehyde was used to immobilize BMSCs cells and then cells were incubated with 0.1% Triton X-100 for 20 min, subsequently, the blocking with 5% BSA/PBS. And then, the cells cocultured with anti-iNOS (ab178945, Abcam) and anti-CD206 antibodies (24595, CST) at 4°C overnight, respectively. Next, the same co-incubation with goat anti-rabbit IgG H&L 1 h and the cells were stained with DAPI and phalloidin. Finally, imaged under a fluorescence microscope and analyzed the results using ImageJ software.

### 2.14 Construction and treatment of osteoarthritis model

All the following animal experiments were conducted in accordance with the protocols approved by the Experimental Animal Ethics Committee of Jiaxing University School (JUMC2021-125). Twenty male Sprague Dawley rats (8 weeks old, weighing 200–250 g) were randomly divided into five groups. Anesthesia in rats were induced under the anesthesia machine with isoflurane at a concentration of 4% and maintained with isoflurane at a concentration of 1%, except the rats in the control group. The medial aspect of the knee joint of the right limb was exposed, and a medical incision was made after disinfection. Then, a rat model was constructed by surgical destabilization of the medial meniscus (DMM). One week after surgery, each group was intra-articular injected with PBS, HM, Mg-PDA@HM and DIC/Mg-PDA@HM (50 μL), and the control group was left untreated. Injections were maintained once a week for 8 weeks.

### 2.15 X-ray imaging and MRI system image analysis

After 8 weeks of treatment, the right knee joints of all rats were analyzed by X-ray radiography. Joint space width was assessed by collecting side view radiographs of the collected joints. Similarly, the right knee joint of living rats was examined by magnetic resonance imaging (MRI) system (Aspect-M3.0) to analyze the regeneration of articular cartilage.

### 2.16 Histopathological and immunohistochemical evaluation

After 8 weeks of treatment and complete imaging, rats were sacrificed by intravenous injection of pentobarbital sodium (100 mg/kg) and their right knee joints were isolated for paraffin embedding to perform histopathological and immunohistochemical assessment. Next, paraffin sections were made for H&E staining and Safranin O/fast green staining. On this basis, the paraffin sections with histopathological staining were scored according to the OARSI standard to assess the extent and depth of joint surface cartilage lesions, and the glycosaminoglycan content was measured by ImageJ software. For immunohistochemical evaluation, the expression of col-II was determined on knee joint sections, and the results were analyzed by ImageJ software.

### 2.17 Statistical analysis

The data were analyzed by GraphPad Prism software. All results were expressed as mean ± standard deviation (SD), all data are analyzed by one-way ANOVA with tukey’s multiple comparisons. ns means no significance, *p < 0.05, **p < 0.01, ***p < 0.001, ****p < 0.0001.

## 3 Results and discussions

### 3.1 Preparation and characterization of Mg-PDA and DIC/Mg-PDA nanoparticles

**FIGURE 1:**
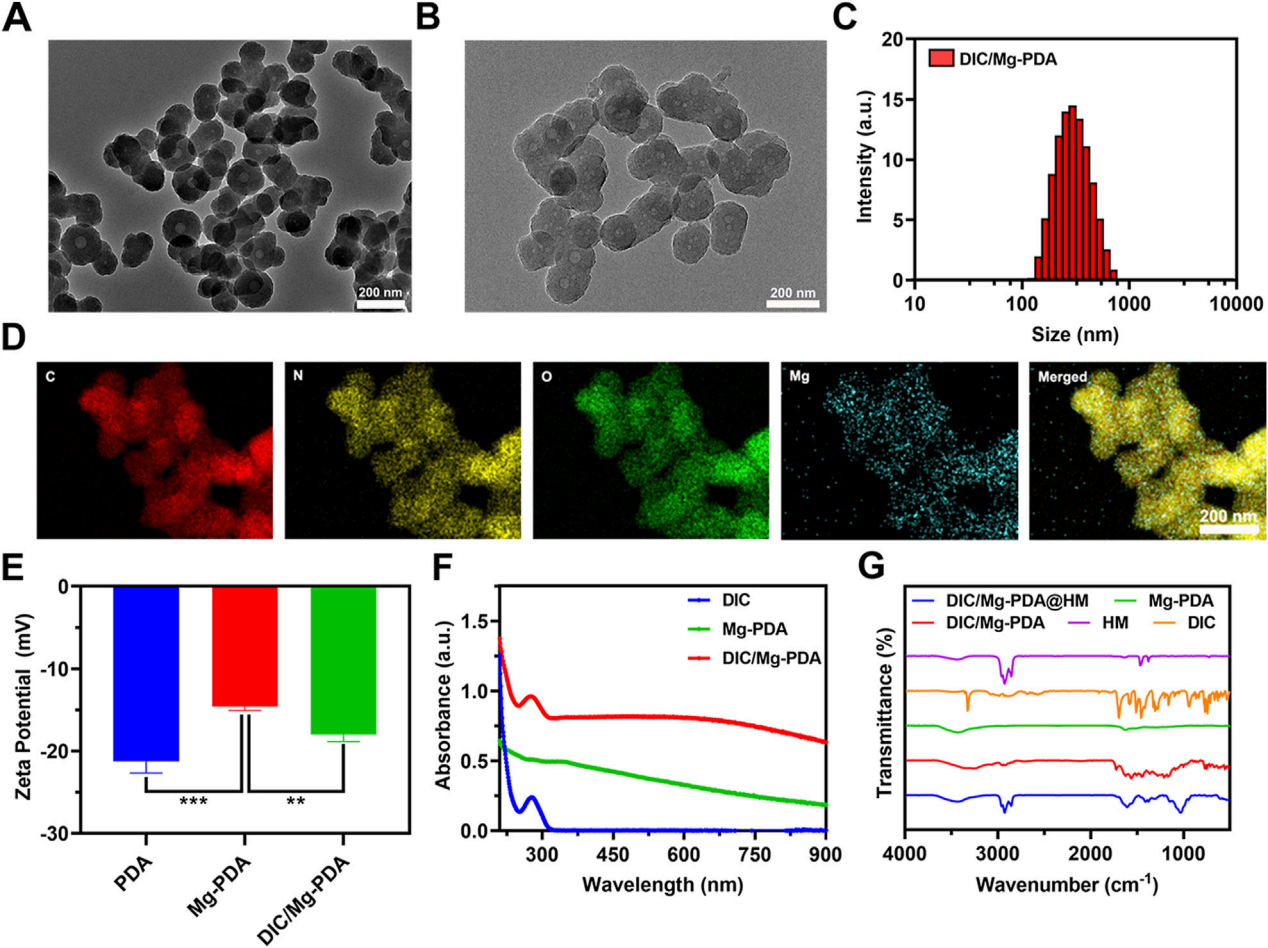
Characterization of DIC/Mg-PDA. **(A)** TEM image of Mg-PDA. **(B)** TEM image of DIC/Mg-PDA. **(C)** Particle size distribution of DIC/Mg-PDA. **(D)** EDS mapping of DIC/Mg-PDA. **(E)** Zeta potential analysis of PDA, Mg-PDA, and DIC/Mg-PDA (n = 3). **(F)** Absorbance of DIC, Mg-PDA, DIC/Mg-PDA. **(G)** FTIR spectra of HM, DIC, Mg-PDA, DIC/Mg-PDA, DIC/Mg-PDA@HM. All the data are presented as mean ± SD, one-way ANOVA with Tukey’s multiple comparisons test, ns means no significance, *p < 0.05, **p < 0.01, ***p < 0.001.

Mg-PDA NPs are innovatively synthesized based on the PDA preparation method reported previously (Liu S. et al., 2023). Briefly, dopamine firstly chelated with Mg^2+^ to form a complex, followed by self-polymerization under the alkaline conditions provided by Tris-HCl to produce Mg^2+^-chelating polydopamine nanoparticles, referred as Mg-PDA NPs. It was found that doping with varying ratios of dopamine and Mg^2+^, nanoparticles with different particle sizes can be formed. As shown in Supplementary Table S1, when the molar ratio of dopamine and Mg^2+^ was 25:1, the smallest size of Mg-PDA NPs was obtained as 232.3 ± 3.4 nm (Supplementary Figure S1), which also exhibited a uniform size with a PDI lower than 0.1. Transmission electron microscopy (TEM) image also showed a homogeneous spherical structure with the size of 162 ± 8 nm (Figure 1A). And the zeta potential of Mg-PDA was increased from −21.8 ± 1.2 mV to −14.5 ± 0.6 mV as compared to PDA (Figure 1E), indicating the successful construction of ideal nanocarriers.

Diclofenac, as a common nonsteroidal anti-inflammatory drug, is widely used in the treatment of OA (da Costa et al., 2021; Li M. et al., 2024). Based on previous reports, we successfully prepared functional nanoparticles DIC/Mg-PDA by activating the carboxyl group in DIC via EDC/NHS and combining it with the amino group on dopamine to form an amide bond (Chen et al., 2023). Under TEM (Figure 1B), we observed that DIC/Mg-PDA remains a homogeneous spherical structure with a size of 190.3 ± 5.1 nm. And dynamic light scattering (DLS) indicated that DIC/Mg-PDA exhibited a size of 292.7 ± 12.5 nm (Figure 1C), indicating that the grafting of DIC only slightly increased the size of particles. In order to further demonstrate the successful construction of the nanoparticles, energy dispersive X-ray spectroscopy (EDS) examination was performed. As shown in Figure 1D, the elements of C, N, O and Mg are uniformly distributed in DIC/Mg-PDA. Since the zeta potential of DIC is negative, the zeta potential of the composite nanoparticle DIC/Mg-PDA is reduced to −17.7 ± 1.2 mV (Figure 1E). This indirectly proves the successful preparation of composite nanoparticles. By measuring the UV- Vis absorption spectra of DIC, Mg-PDA and DIC/Mg-PDA (Figure 1F), it can be found that there was a maximum characteristic absorption peak of DIC at 280 nm for DIC/Mg-PDA, and the drug loading ratio of DIC was calculated to be 7.51% ± 0.21% by using the metric. In addition, the 1,602 cm^−1^ characteristic peak of DIC/Mg-PDA in fourier transform infrared (FTIR) spectroscopic detection was attributed to the formation of amide bonds between dopamine and DIC via amide reaction, and the simultaneous appearance of the characteristic peaks of DIC and Mg-PDA once again proved that the composite nanoparticles were successfully constructed (Figure 1G) (Chen et al., 2023).

### 3.2 Preparation and characterization of HM and DIC/Mg-PDA@HM

**FIGURE 2:**
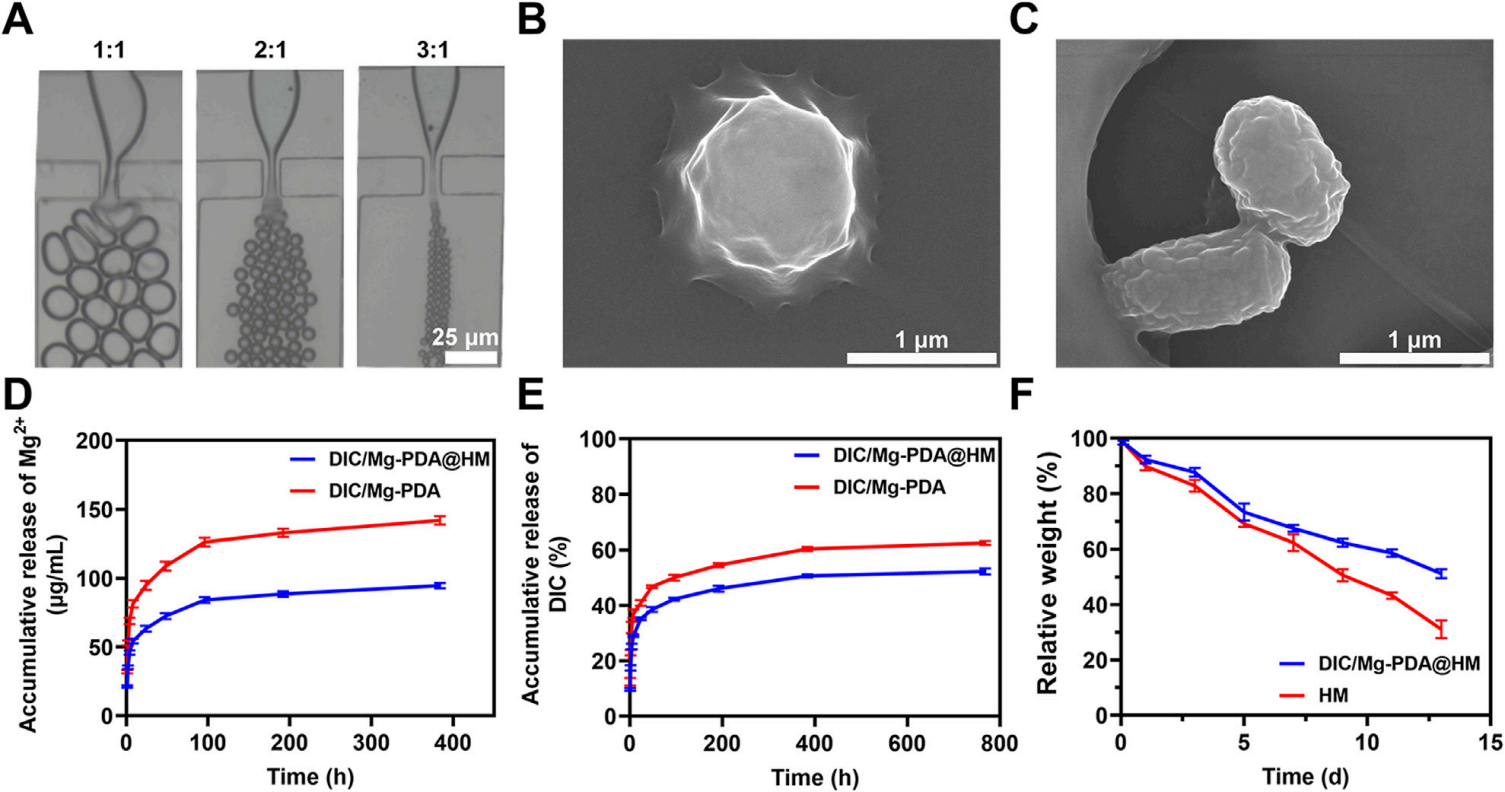
Characterization of hydrogel microspheres. **(A)** Microscopic images of microgels in microfluidic chips at different hydro-oil flow rates (1:1, 1:2 and 1:3). **(B)** SEM images of HM. **(C)** SEM images of DIC/Mg-PDA@HM. **(D)** Release behavior of Mg^2+^ from DIC/Mg-PDA and DIC/Mg-PDA@HM in 1X PBS solution. **(E)** Release behavior of DIC from DIC/Mg-PDA and DIC/Mg-PDA@HM in 1X PBS solution. **(F)** Degradation behavior of HM and DIC/Mg-PDA@HM.

HAMA hydrogel microspheres (HM) were prepared by photopolymerization based on microfluidic technology (Lei et al., 2022). As shown in Figure 2A, in a microfluidic device, varying the flow rate ratio of the aqueous phase (HAMA, DIC/Mg-PDA, LAP) and the oil phase (mineral oil, Span 80) can produce hydrogel microspheres with different sizes. In order to better achieve the injectability of the composite microspheres, a flow rate ratio of 1:3 between the aqueous and oil phases was selected in this study. The morphologies of lyophilized HM and DIC/Mg-PDA@HM were measured under scanning electron microscopy (SEM). The lyophilized HM were relatively rounded and spherical (Figure 2B), whereas the lyophilized DIC/Mg-PDA@HM had an inhomogeneous relatively rough appearance with a size of 1.2 ± 0.21 μm (Figure 2C). In order to verify the successful construction of DIC/Mg-PDA@HM, FTIR spectroscopic detection was performed. As shown in Figure 1G, the characteristic peaks of DIC/Mg-PDA and HMs appeared simultaneously in the spectrum of DIC/Mg-PDA@HM, which once again verified the successful preparation of composite hydrogel microspheres. Finally, to evaluate the distribution of nanoparticles within the hydrogel microspheres, we prepared fluorescent labeling hydrogel microspheres by conjugating ICG to the surface of the nanoparticles. As show in Supplementary Figure S2, the red fluorescent nanoparticles can be directly observed throughout the microspheres.

### 3.3 Drug release and degradation

In order to evaluate the release behavior of metal ions and drugs from DIC/Mg-PDA@HM *in vitro*, the release profile was investigated in PBS. As shown in Figure 2D, Mg^2+^ was in a burst release during the initial 4 h, and the subsequent growth rate slowed down into a relatively smooth release phase. Possibly due to the effect of the encapsulation of the hydrogel matrix and the nature of the nanomaterials, the total release of Mg^2+^ from DIC/Mg-PDA decreased from 144.03 ± 2.05 μg/mL to 98.52 ± 1.73 μg/mL compared to that from DIC/Mg-PDA@HM. In Figure 2E, the release of DIC was similar to that of Mg^2+^, with the total release rate of DIC decreasing from 62.12% ± 2.36% to 51.25 ± 1.95. Consequently, DIC/Mg-PDA@HM performed much better in terms of slow and controlled drug release. Then, to investigate the biodegradation performance of microspheres, HM and DIC/Mg-PDA@HM were soaked in type I collagenase-containing solution. As shown in Figure 2F, HM and DIC/Mg-PDA@HM degraded 51.16% ± 1.17% and 30.51% ± 2.78% at 14 days, and the collagenase solution well mimicked the physiological miroenvironment of the joints. Thus, DIC/Mg-PDA@HM demonstrated a good biodegradability.

### 3.4 *In vitro* biocompatibility of DIC/Mg-PDA@HM

**FIGURE 3:**
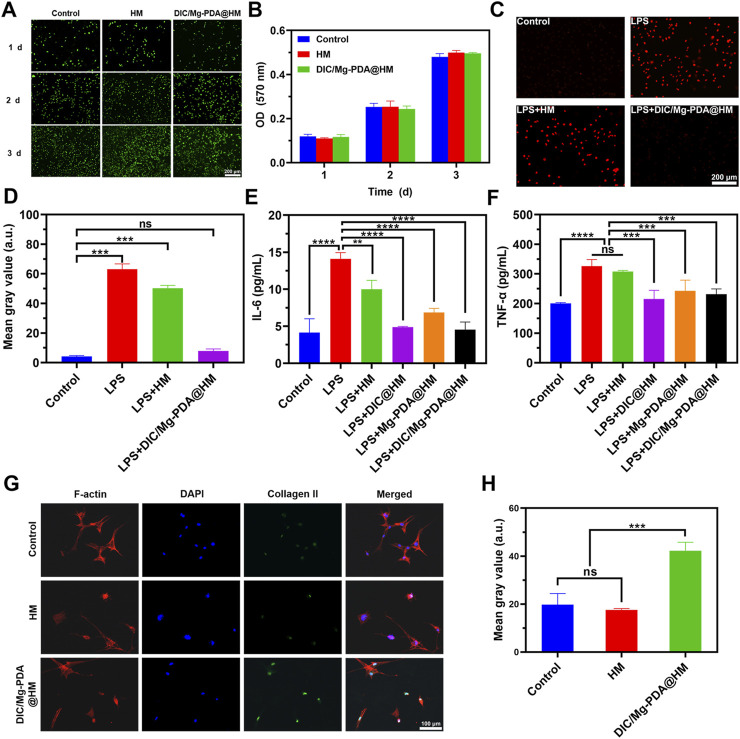
*In vitro* cytotoxicity, cellular behavior of hydrogel microspheres against ATDC cells and against BMSCs. **(A)** Live/dead staining results of ATDC5 cells in Control, HM, and DIC/Mg-PDA@HM groups at 1, 2, and 3 days. **(B)** MTT results of ATDC5 cells in Control, HM, DIC/Mg-PDA@HM groups at 1, 2 and 3 days (n = 3). **(C)** Dihydroethidium (DHE) staining results of Control, LPS, LPS+HM, LPS+DIC/Mg-PDA@HM. **(D)** ImageJ analysis of DHE staining (n = 3). Levels of **(E)** IL-6 and **(F)** TNF-α secreted by ATDC5 cells after PBS, LPS, LPS+HM, LPS+DIC@HM, LPS+Mg-PDA@HM, and LPS+DIC/Mg-PDA@HM treatment (n = 3). **(G)** Immunofluorescence staining results of BMSCs treated in Control, HM, and DIC/Mg-PDA@HM groups. **(H)** ImageJ analysis of fluorescence intensity of collagen II expression (n = 3). All the data are presented as mean ± SD, one-way ANOVA with Tukey’s multiple comparisons test, ns means no significance, *p < 0.05, **p < 0.01, ***p < 0.001, ****p < 0.0001.

*In vitro* experiments, in order to evaluate the biocompatibility of DIC/Mg-PDA@HM, we added HM and DIC/Mg-PDA@HM to the chondrocyte cells (ATDC5 cell line) and examined them with Live/Dead staining assays and MTT assays. As shown in Figure 3A, Most of ATDC5 cells exhibited good bioactivity and a significant increase in cell density during the 3-day culture. Similarly, as show in the MTT results (Figure 3B), the amount of ATDC5 cells gradually increased over time and there was no significant disparities between the groups. In summary, DIC/Mg-PDA@HM demonstrated a good biocompatibility in Live/Dead staining and MTT assays.

### 3.5 *In vitro* cellular uptake

To verify the *in vitro* cellular uptake of DIC/Mg-PDA@HM in chondrocytes, we prepared composite hydrogel microspheres with fluorescence localization by loading indocyanine green (ICG) on the surface of nanoparticles. The cellular uptake was detected by incubating samples with ATDC5 cell line at 6 h and 24 h, respectively. As shown in Supplementary Figure S3, the released reddish fluorescent nanoparticles in DIC/Mg-PDA@HM were successfully uptaken by the cells and distributed around the nucleus, and the fluorescence intensity increased with time. Therefore, the good cellular uptake ability of DIC/Mg-PDA@HM was confirmed.

### 3.6 *In vitro* ROS scavenging and anti-inflammatory assessment

It is well known that low doses of ROS play a crucial role in maintaining cellular homeostasis, and excessive ROS disrupt the original oxidative homeostasis and induce apoptosis (Shi et al., 2023). In this study, the inflammatory microenvironment of OA caused by excessive ROS was simulated by LPS, and intracellular ROS levels were detected using dihydroethidium (DHE). As shown in Figure 3C, after LPS induction, ATDC5 cells produced excessive ROS, which appeared bright red under DHE staining, and was found to be not significantly different from the results of HM group by quantification (Figure 3D). While the ROS level was significantly decreased under the effect of DIC/Mg-PDA@HM, and there was no significant difference with the results of normal cells after quantification. The anti-oxidative stress effect of DIC/Mg-PDA@HM was successfully verified.

TNF-α and IL-6, as typical inflammatory factors during OA progression, are often used as indicators of reactive cellular inflammation (Liu L. et al., 2023; Guo et al., 2024). The anti-inflammatory effects of DIC/Mg-PDA@HM were further assessed by detecting the expression of TNF-α and IL-6 through ELISA kits. As shown in Figures 3E, F, the ATDC5 chondrocyte cell line exhibited extremely high levels of TNF-α and IL-6 after incubation with LPS for 24 h, which represented a strong inflammatory response. In the HM group, the inflammatory indexes were reduced, but still maintained at a high level. And in the DIC@HM, Mg-PDA@HM and DIC/Mg-PDA@HM groups, they all showed good anti-inflammatory effects, among which the DIC@HM and DIC/Mg-PDA@HM groups showed better anti-inflammatory effects, and there was no significant difference between the two groups. In conclusion, DIC/Mg-PDA@HM can efficiently alleviate inflammation.

### 3.7 Evaluation of chondrogenic differentiation *in vitro*

Collagen-II plays a crucial role in cartilage tissue regeneration (Moon et al., 2022). Therefore, to assess the cartilage differentiation effect of Mg ions released from DIC/Mg-PDA@HM on BMSCs, we investigated the expression of Col-II on BMSCs, which was used as a representative indicator reflecting cartilage differentiation. As shown in Figure 3G, the green fluorescence intensity of the cells incubated with DIC/Mg-PDA@HM was significantly increased compared with that of the control group, which proved that DIC/Mg-PDA@HM effectively promoted cartilage differentiation. The same conclusion was drawn more intuitively by quantification (Figure 3H).

### 3.8 Effect of DIC/Mg-PDA@HM on macrophage phenotypic transformation

**FIGURE 4:**
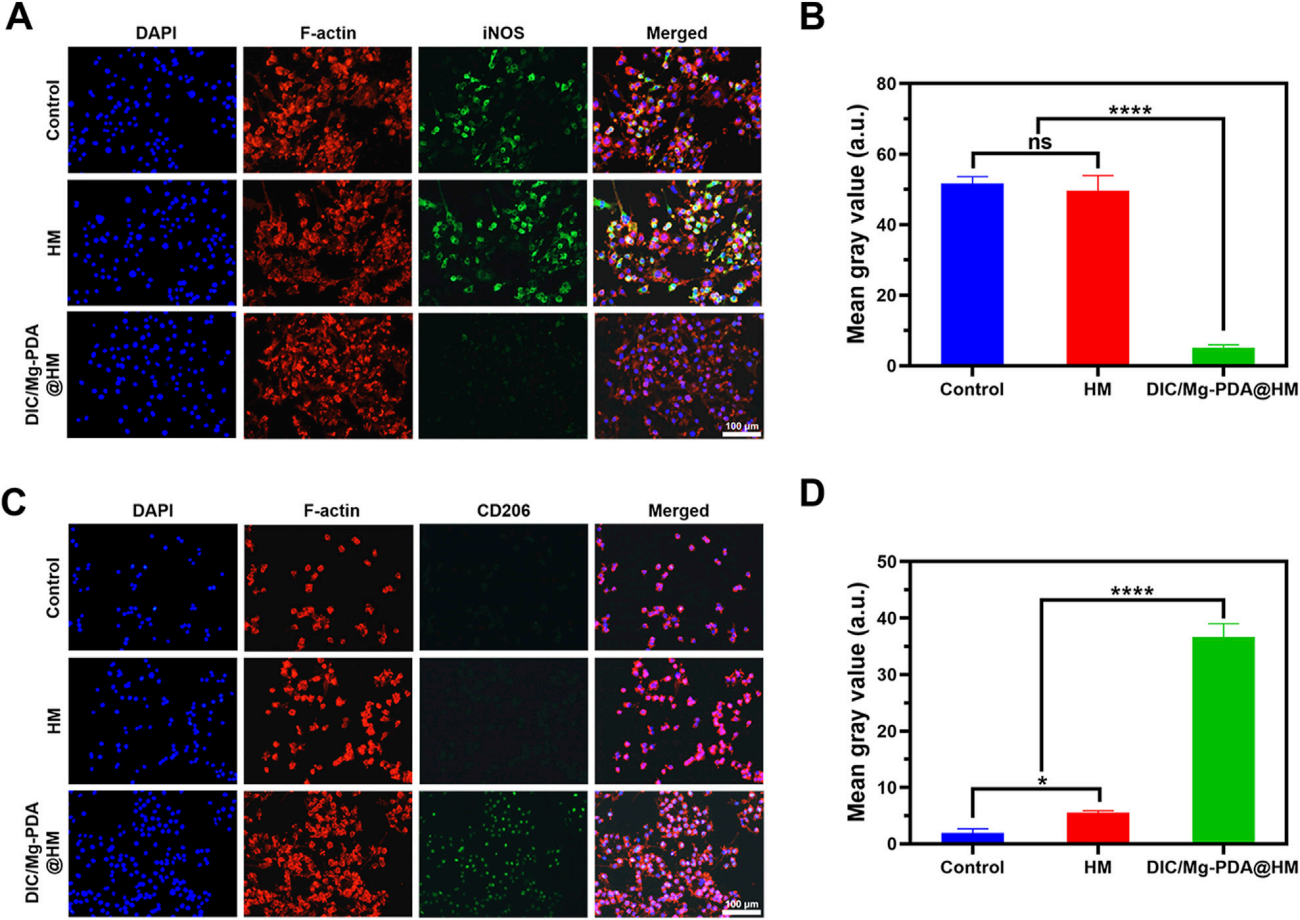
Behavior of hydrogel microspheres on RAW264.7 cells. Immunofluorescence staining results of RAW264.7 treated in **(A, C)** Control, HM and DIC/Mg-PDA@HM groups. M1 marker: iNOS and M2 marker: CD206. ImageJ analysis of fluorescence intensity of iNOS **(B)** and CD206 **(D)** (n = 3). All the data are presented as mean ± SD, one-way ANOVA with Tukey’s multiple comparisons test, ns means no significance, *p < 0.05, **p < 0.01, ***p < 0.001, ****p < 0.0001.

To assess the effect of Mg^2+^ released from DIC/Mg-PDA@HM on macrophage phenotypic transformation, we simulated the OA inflammatory microenvironment *in vitro* by similarly adding LPS to the macrophage cell line RAW264.7, followed by 24 h incubation with DIC/Mg-PDA@HM. Then, we investigated the macrophage phenotypic by determining the expression of the M1 macrophage surface marker (iNOS) and M2 macrophage surface marker (CD206) (Huang et al., 2024). As shown in Figures 4A, B, iNOS levels expressed in Control and HM groups were significantly higher than that in DIC/Mg-PDA@HM after incubation with LPS, suggesting that DIC/Mg-PDA@HM could inhibit the transformation of macrophages to the M1 phenotype. In Figures 4C, D, the level of CD206 was the significantly highest in the DIC/Mg-PDA@HM group as compared to other groups, revealing its promotion of macrophage transformation to the M2 phenotype. Thus, DIC/Mg-PDA@HM possessed a good immune microenvironment regulation.

### 3.9 *In vivo* therapeutic efficacy in osteoarthritis

**FIGURE 5:**
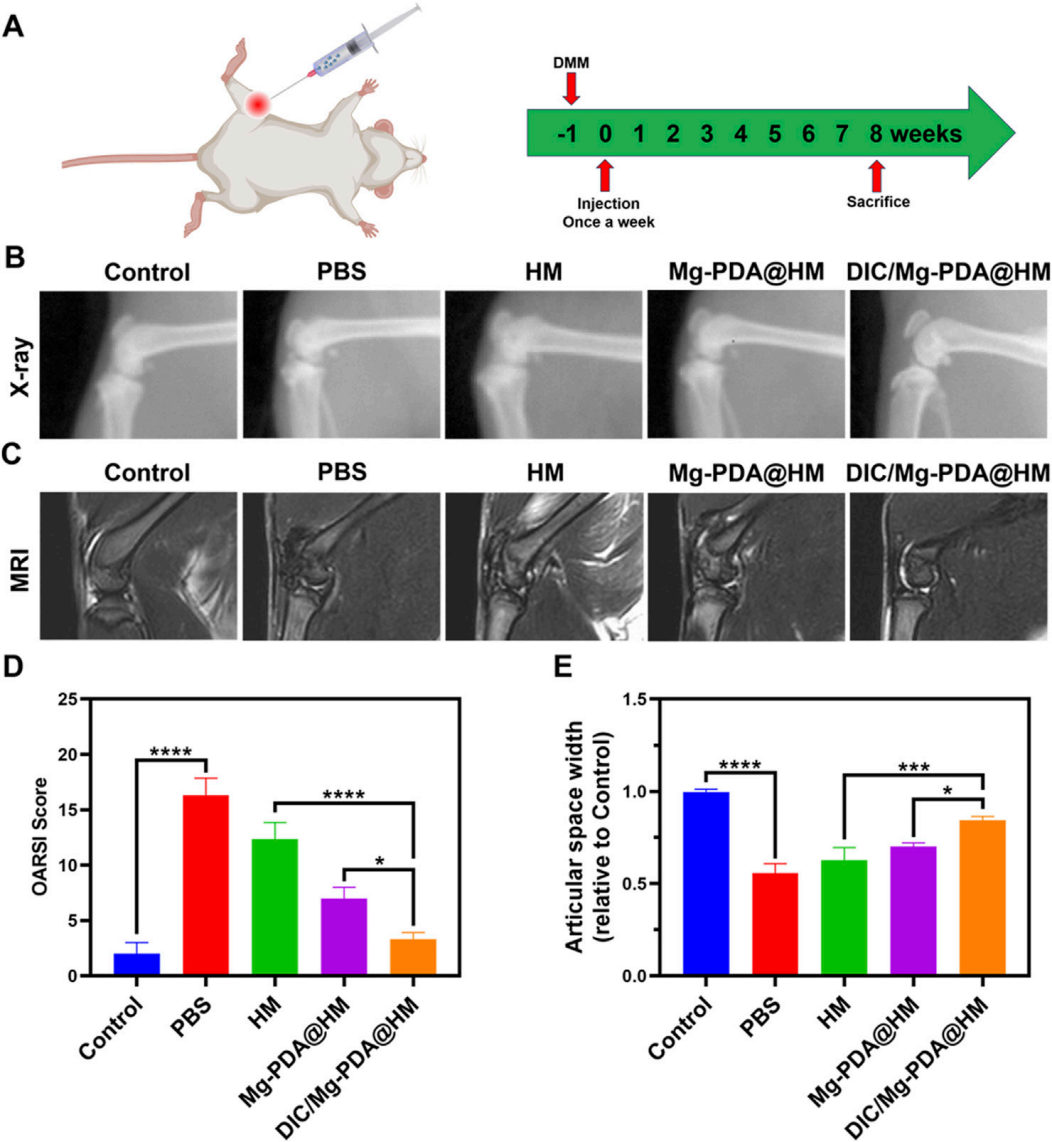
*In vivo* therapeutic effect of DIC/Mg-PDA@HM. **(A)** Therapeutic regimen of intra-articular injection in OA rats. **(B)** X-ray images taken 8 weeks after weekly injections of PBS, HM, Mg-PDA@HM and DIC/Mg-PDA@HM in each group of rats. **(C)** MRI scans of rats in each group taken 8 weeks after weekly injections of PBS, HM, Mg-PDA@HM and DIC/Mg-PDA@HM. **(D)** OARSI scores of articular cartilage in different groups. **(E)** Relative joint space width in different groups. All the data are presented as mean ± SD, one-way ANOVA with Tukey’s multiple comparisons test, ns means no significance, *p < 0.05, **p < 0.01, ***p < 0.001, ****p < 0.0001.

*In vitro* studies, DIC/Mg-PDA@HM demonstrated a good therapeutic potential for OA, and in order to further evaluate the therapeutic effect *in vivo*, we constructed a rat model of osteoarthritis by the surgical destabilization of the medial meniscus (DMM) (Deng et al., 2024; Wei et al., 2024). After 1 week, the joint cavities of the different groups of rats were injected once a week with PBS, HM, Mg-PDA@HM, and DIC/Mg- PDA@HM for a total of 8 weeks (Figure 5A). Finally, X-ray imaging, Magnetic Resonance Imaging (MRI), and immunohistochemical staining were conducted to evaluate the therapeutic effect on OA (Wei et al., 2024).

After 8 weeks of treatment, the morphology of the joints was assessed using X-rays (Figure 5B). Severe degeneration of articular cartilage, joint narrowing, and bone redundancy formation in the PBS and HM groups compared to the control group, which indicating a significant progression of OA. In contrast, the joints of the rats in the Mg-PDA@HM and DIC/Mg-PDA@HM groups showed attenuation of OA progression, with the DIC/Mg-PDA@HM group demonstrating a better therapeutic effect in terms of joint morphology. The same performance was also reflected in the relative joint gap widths of the groups (Figure 5E), compared with the control group, the rats in the PBS and HM groups had extensive formation and sclerosis of intra-articular osteophytes, resulting in a significantly narrower gap. Whereas the narrowing of the joint gap in the rats in the Mg-PDA@HM and DIC/Mg-PDA@HM groups was mitigated, and the DIC/Mg-PDA@HM group demonstrated a better joint gap width. Therefore, the multifunctional hydrogel microspheres demonstrated their excellent OA therapeutic effects *in vivo*, which may contribute to the synergistic effect between their immunomodulation of anti-inflammatory and chondrogenic differentiation.

In addition, we performed MRI in the rat knee joint to reflect the progression of OA. As shown in Figure 5C, the normal knee cartilage showed smooth, continuous and homogeneous signals on the T2-weighted sequence. In contrast, the articular cartilage in the PBS and HM groups clearly exhibited rough, interrupted and mixed signals, suggesting severe cartilage degeneration. However, the articular cartilage signals in the Mg-PDA@HM and DIC/Mg-PDA@HM groups were relatively better. Among them, DIC/Mg-PDA@HM performed the best, presenting a signal similar to that of normal cartilage. It showed its excellent ability of cartilage repairing *in vivo*.

**FIGURE 6:**
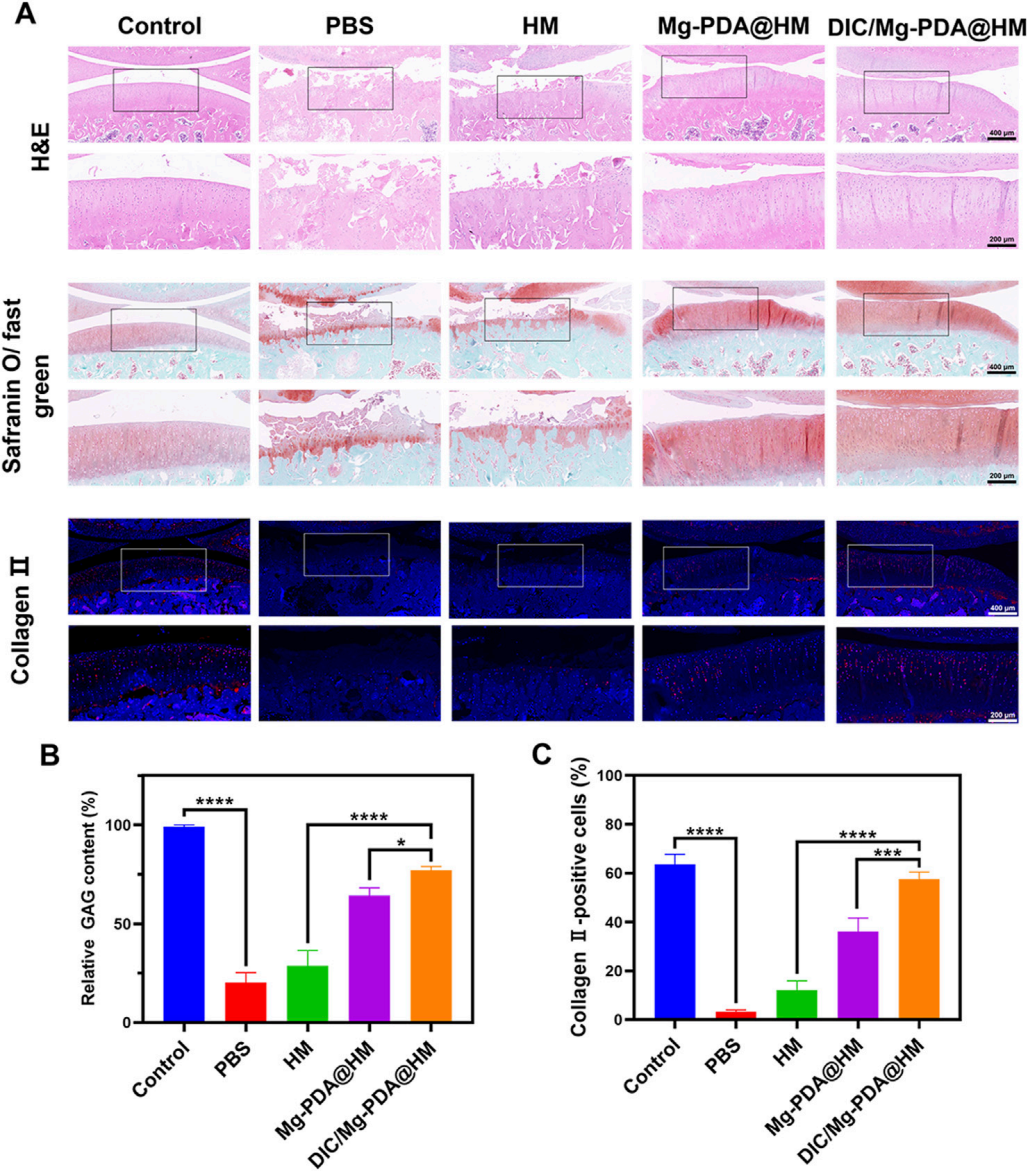
DIC/Mg-PDA@HM attenuated the progression of OA in the model. **(A)** Histopathological and immunohistochemical images of different groups. **(B)** The level of GAG in different groups (n = 3). **(C)** The level of Collagen-II in different groups (n = 3). All the data are presented as mean ± SD, one-way ANOVA with Tukey’s multiple comparisons test, ns means no significance, *p < 0.05, **p < 0.01, ***p < 0.001, ****p < 0.0001.

To further assess the cartilage-repairing ability *in vivo*, we also performed histological and immunohistochemical assessments. In hematoxylin and eosin (H&E) staining and Safranin O-fast green staining (Figure 6A), the surface of cartilage layer was significantly eroded in the PBS and HM groups, whereas the DIC/Mg-PDA@HM group showed an intact cartilage layer similar to that of normal joints. In addition, Safranin O-fast green staining also suggested that the DIC/Mg-PDA@HM group received a relatively low score on the OARSI score (Figure 5D), which probably attributed to the excellent anti-inflammatory and chondrogenic differentiation effects of DIC/Mg-PDA@HM. Moreover, compared with the control group, there was a significant increase in glycosaminoglycan (GAG) content in the rats injected with DIC/Mg-PDA@HM (Figure 6B). Once again, its role in maintaining cartilage was demonstrated. Subsequently, we assessed the level of collagen- II using immunofluorescence assays (Figure 6C). Compared with the control group, the level of collagen II was reduced in all groups except the DIC/Mg-PDA@HM group, which possessed the highest expression of collagen II, suggesting a better OA treatment effect. In addition, to verify the biotoxicity of the composite microspheres, heart, liver, spleen, lung and kidney samples were collected from each group of rats 8 weeks after material injection, subsequently, samples were subjected to tissue sectioning and H&E staining (Supplementary Figure S4). The results showed that the materials had no significant deleterious effects on the organs of the rats after implantation. All these above demonstrated a promising way to achieve cartilage regeneration in OA therapy.

## 4 Conclusion

Comprehensive and systematic treatment of OA is a great challenge in clinical practice due to its complex pathogenesis and poor tissue self-repairing conditions. In this study, we developed a novel multifunctional injectable hydrogel micro-sphere DIC/Mg-PDA@HM with micro-nano structure for OA therapy, which reversed the suppressed microenvironment and promoted cartilage regeneration. After injected into the joint cavity, the hydrogel microsphere DIC/Mg-PDA@HM was able to not only effectively downregulate inflammatory factors and modulate macrophage phenotypic transformation, but also promote chondrogenic differentiation and achieve cartilage regeneration. In conclusion, these multifunctional injectable hydrogel microspheres DIC/Mg-PDA@HM may provide a novel and promising strategy for the treatment of OA.

## Data Availability

The original contributions presented in the study are included in the article/Supplementary Material, further inquiries can be directed to the corresponding authors.
