# Supplementary material for: Metal ion-crosslinking multifunctional hydrogel microspheres with inflammatory immune regulation for cartilage regeneration
Source: Front Bioeng Biotechnol. 2025 Jan 28;13:1540592. doi: 10.3389/fbioe.2025.1540592 (PMC11810939; doi:10.3389/fbioe.2025.1540592)
Supplement: Supplementary file 1 [file DataSheet1.docx]

**Supporting Information**

**Metal ion-crosslinking multifunctional hydrogel microspheres with inflammatory immune regulation for cartilage regeneration**

**Zhuoming Xu^1,2,^** †**, Jun Ma^2,^** †**, Hanyin Hu^1,2,^** †**, Jintao Liu^1,2^, Haiyang Yang^1,2^, Jiayi Chen^2^, Hongwei Xu^2^, Xinyu Wang^3^, Huanhuan Luo^2^,*, Gang Chen^2^,***

^1^ Jiaxing University Master Degree Cultivation Base, Zhejiang Chinese Medical University, 310000, China.

^2^ Department of Orthopaedics, Jiaxing Key Laboratory of Basic Research and Clinical Translation on Orthopedic Biomaterials, The Second Affiliated Hospital of Jiaxing University, 1518 North Huancheng Road, Jiaxing 314000, P. R. China.

^3^ Department of Radiology, The Second Affiliated Hospital of Jiaxing University, 1518 North Huancheng Road, Jiaxing 314000, P. R. China.

† These authors have contributed equally to this work and share first authorship

*** Correspondence:**Gang Chen ([adcyy@aliyun.com](mailto:adcyy@aliyun.com));

Huanhuan Luo (luohh@zjxu.edu.cn );

| **Mg^2+^ : Dopamine** | **Size (nm)** | **PDI** |
| --- | --- | --- |
| **1:5** | **337.1±11.4** | **0.166±0.025** |
| **1:10** | **298.8±6.1** | **0.103±0.016** |
| **1:25** | **232.3±3.4** | **0.077±0.009** |
| **1:50** | **315.2±7.5** | **0.049±0.019** |

**Table S1.** The sizes of Mg-PDA NPs prepared with different Mg^2+^ concentrations.


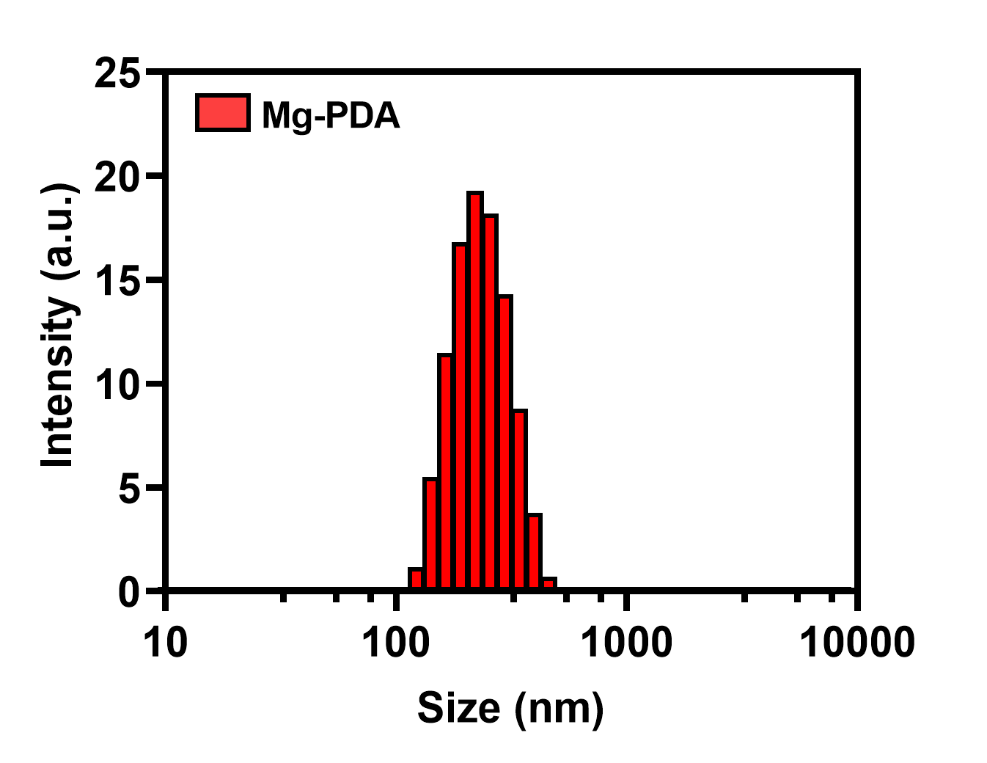


**Figure S1**. Particle size distribution of Mg-PDA


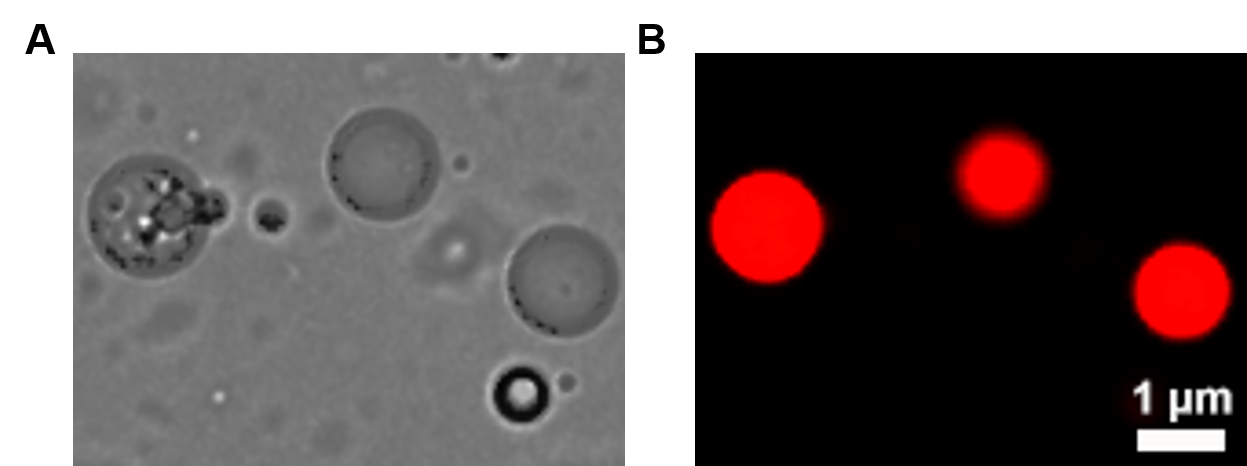


**Figure S2**. Co-localization test of DIC/Mg-PDA@HM.


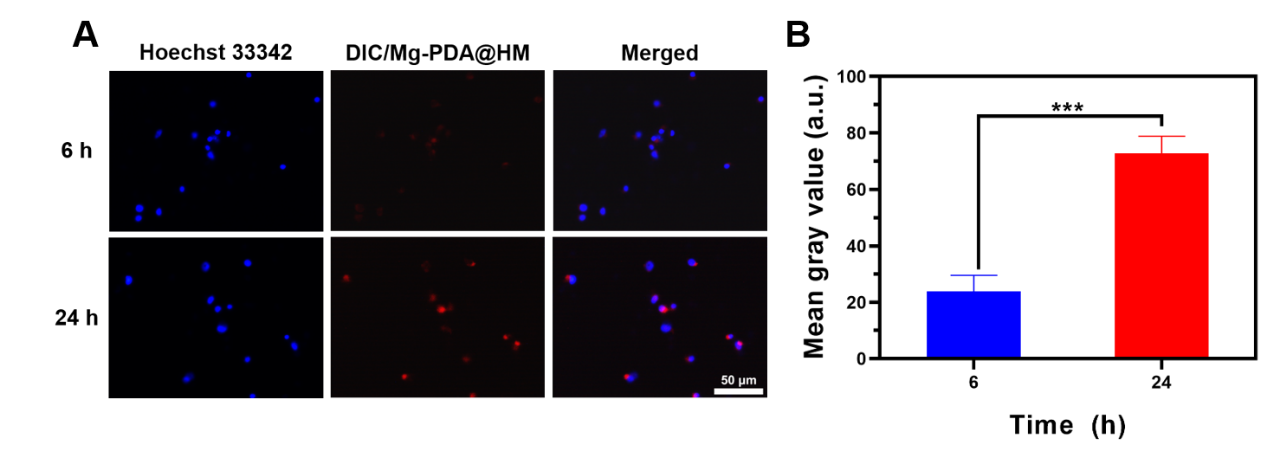
**Figure S3**. (A) The fluorescent microscope images of ATDC5 cells treated with DIC/Mg-PDA@HM. (B) Image J analysis of the cellular uptake (DIC/Mg-PDA@HM was labeled by ICG, *n* = 3, ****P* < 0.001).


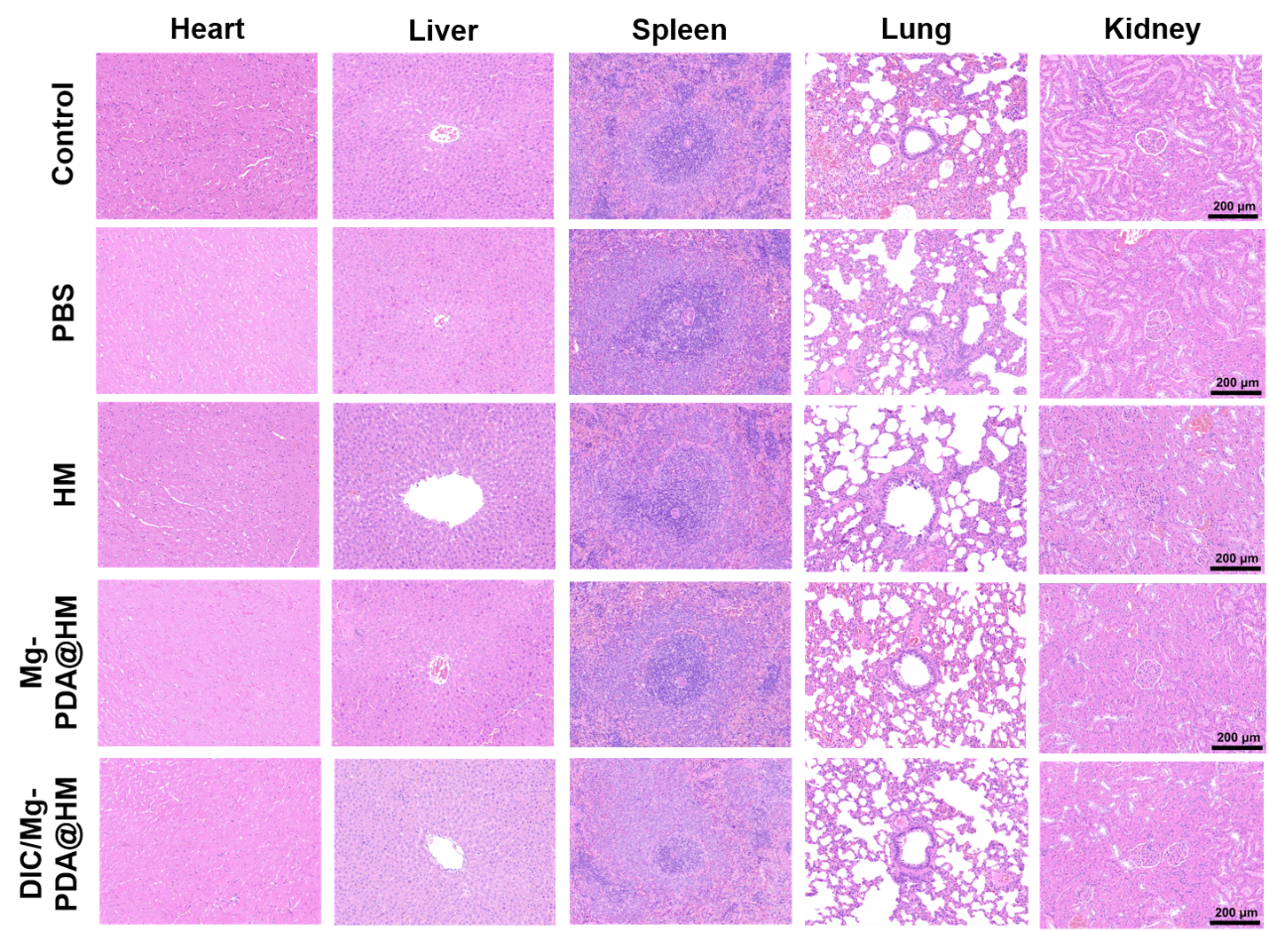


**Figure S4**. H&E staining of the heart, liver, spleen, lung and kidney tissues dissected from the mice treated with various groups.
